# Supplementary material for: Expanding risks: Medicaid expansion and data security
Source: PLoS One. 2025 Jul 24;20(7):e0307015. doi: 10.1371/journal.pone.0307015 (PMC12289059; doi:10.1371/journal.pone.0307015)

For Expanding Risks: Medicaid Expansion and Data Security

***Supporting Information***

**This PDF file includes:**

S1 Appendix: Robustness Tests

S2 through S7: Exhibits A1 – A7

# S1 Appendix: Robustness Tests

In what follows, we provide a brief overview of additional empirical tests to ensure the robustness of our findings.

## Placebo Tests

As discussed in Bertrand et al (1), one consistent issue with phased implementation difference in difference estimations is the possibility for serial correlation in the standard errors. This is concerning, because if there is serial correlation in the standard errors they will become artificially deflated. This subsequently increases the possibility of finding significant results purely by chance. A popular check to safeguard against this possibility is a random implementation test (2, 3). To execute this test, we randomly apply the treatment to an equal proportion of the sample as was originally treated, replicate the estimation, and store the coefficient. This process is replicated 1000 times, allowing us to determine the probability that the estimated effect will manifest purely by chance.

Results are in Exhibit A2. Three things are immediately apparent from the results. First, the mean placebo effect is near zero, suggesting that the randomization is effective. Second, the standard deviation straddles zero with t-values ranging between [0.09] and [0.035] (i.e., the p-value is never close to significant). Third, and finally, calculation of the p-value of the observed effect, as compared with the pseudo-treatment is (p<0.0001) in all estimations, suggesting the probability of arriving at any of the observed coefficients, even the insignificant ones, purely by chance is vanishingly small. Thus, the probability of estimating the observed effect by chance is negligible and can be dismissed outright.

## Phased Implementation Concerns

While canonical representations of the DID (see Card and Krueger (4)) relied on a single treatment which compared two groups, pre- and post, econometric investigations have recently gravitated towards phased difference in difference comparisons where different groups receive the treatment at different times. Yet, recent advances in the econometrics literature have raised the possibility that these phased implementations may result in inverse weighting problems and yield biased results (5-7). This is because, during a phased implementation, the effect shifts, becoming a weighted average of early vs. late treatment, treated vs. never treated, and so forth. As a result, the individual weights of the components of the estimate may invert, yielding a biased coefficient which can even invert the sign of the coefficient, notably when the individual treatments differ in size and grow with time.

The econometrics literature suggests several possible solutions to this problem. We employ three. First, we execute a Goodman-Bacon (6) decomposition, wherein the weights of each component are calculated directly. Second, we replicate the estimations dropping all treated states which were *not* treated in 2014 (i.e., the dominant year of implementation). As a result, the estimated coefficient becomes a simple DID, with treatment exclusively occurring in 2014 and the control group being non-Medicaid expanding states. Finally, we implement a Callaway-Sant’Anna (7) doubly-robust difference in difference (CSDID) estimator. The CSDID estimator allows for treatment heterogeneity by calculating the treatment effect across all possible timing and control combinations, which can then be aggregated.

Results of the Goodman-Bacon (6) decomposition are in Exhibit A3, and indicate no inverse weighting issues of note, i.e. all weights are positive for both identity theft and fraud. This suggests limited evidence of an inverse weighting problem. Results of the binned 2014 estimates are in Exhibit A4. As can be seen, the results become stronger, suggesting a strong and significant effect on fraud in Column 2, with no material effect on identity theft (Column 1). Finally, results of the Callaway and Sant’Anna (7) are in Exhibit A5 (Identity Theft) and A6 (Fraud). These figures aggregate the group-time average treatment effects calculated by the CSDID estimator. Results are consistent with the conclusions drawn above—there appears to be a significant reduction on the rate of fraud (with an aggregated treatment effect of -0.063, p = 0.016). Similarly, the results for identity theft yield a non-significant aggregated treatment effect of 0.032 with a confidence interval straddling zero, p = 0.66, along with a possible pre-trend. Taken in sum, these results corroborate the material effect of Medicaid expansion on fraud, with no observed effect on identity theft.

# References

1. Bertrand M, Duflo E, Mullainathan S. How much should we trust differences-in-differences estimates? The Quarterly Journal of Economics. 2004;119(1):249-75.

2. Burtch G, Carnahan S, Greenwood BN. Can You Gig it? An Empirical Examination of the Gig-Economy and Entrepreneurial Activity. Management Science. 2018;64(12):5497-520.

3. Greenwood B, Agarwal R. Matching Platforms and HIV Incidence: An Empirical Investigation of Race, Gender, and Socio-Economic Status. Management Science. 2016;62(8):2281-303.

4. Card D, Krueger AB. Minimum wages and employment: A case study of the fast food industry in New Jersey and Pennsylvania. National Bureau of Economic Research; 1993. Report No.: 0898-2937.

5. Baker A, Larcker DF, Wang CC. How Much Should We Trust Staggered Difference-In-Differences Estimates? Available at SSRN 3794018. 2021.

6. Goodman-Bacon A. Difference-in-differences with variation in treatment timing. National Bureau of Economic Research; 2018. Report No.: 0898-2937.

7. Callaway B, Sant’Anna PH. Difference-in-differences with multiple time periods. Journal of Econometrics. 2020.

**S2 Exhibit A1:** Treatment Schedule

| **State** | **Expansion Year** |  | **State** | **Expansion Year** |
| --- | --- | --- | --- | --- |
| Connecticut | 2012 |  | West Virginia | 2014 |
| Arizona | 2014 |  | Alaska | 2015 |
| Arkansas | 2014 |  | Indiana | 2015 |
| California | 2014 |  | Pennsylvania | 2015 |
| Colorado | 2014 |  | Louisiana | 2016 |
| Delaware | 2014 |  | Montana | 2016 |
| District of Columbia | 2014 |  | Maine | 2018 |
| Hawaii | 2014 |  | Idaho | 2019 |
| Illinois | 2014 |  | Virginia | 2019 |
| Iowa | 2014 |  | Nebraska | 2020 |
| Kentucky | 2014 |  | Utah | 2020 |
| Maryland | 2014 |  | Missouri | 2021 |
| Massachusetts | 2014 |  | Oklahoma | 2021 |
| Michigan | 2014 |  | Alabama | - |
| Minnesota | 2014 |  | Florida | - |
| Nevada | 2014 |  | Georgia | - |
| New Hampshire | 2014 |  | Kansas | - |
| New Jersey | 2014 |  | Mississippi | - |
| New Mexico | 2014 |  | North Carolina | - |
| New York | 2014 |  | South Carolina | - |
| North Dakota | 2014 |  | South Dakota | - |
| Ohio | 2014 |  | Tennessee | - |
| Oregon | 2014 |  | Texas | - |
| Rhode Island | 2014 |  | Wisconsin | - |
| Vermont | 2014 |  | Wyoming | - |
| Washington | 2014 |  |  |  |
| States with a “-“ indicator have yet to expand their Medicaid coverage. | | | | |

**S3 Exhibit A2:** Effect of Medicaid Expansion on Breaches and Record Theft as Defined by the Privacy Rights Clearinghouse (Sample Restricted to 2010 Forward)

|  | (1) | (2) |
| --- | --- | --- |
| Estimator | OLS | OLS |
| Dependent Variable | ln(Breaches) | ln(Records) |
|  |  |  |
| Treatment | -0.144* | -1.125** |
|  | (0.0766) | (0.549) |
|  |  |  |
| State FE | Yes | Yes |
| Year FE | Yes | Yes |
| Observations | 510 | 510 |
| R-squared | 0.201 | 0.133 |
| Number of Groups | 51 | 51 |

Robust standard errors in parentheses (clustered on state)

*** p<0.01, ** p<0.05, * p<0.1

**S4 Exhibit A3:** Placebo Tests

|  | (1) | (2) |
| --- | --- | --- |
| Estimator | PPML | PPML |
| Dependent Variable | Identity Theft | Fraud |
|  |  |  |
| Treatment | -0.0747* | -0.177*** |
|  | (0.0448) | (0.0599) |
| Pseudo-Effect | -0.00220279 | -0.00092596 |
| Pseudo-Std Dev | 0.03359313 | 0.026144293 |
| p-value | p<0.000 | p<0.000 |
| Observations | 765 | 765 |
| Number of Groups | 51 | 51 |
| Robust standard errors in parentheses (clustered on state)  *** p<0.01, ** p<0.05, * p<0.1 | | |

**S5 Exhibit A4:** Weights from the Goodman-Bacon Decomposition

|  | (1) | (2) | (3) | (4) |
| --- | --- | --- | --- | --- |
| Estimator | OLS | OLS | OLS | OLS |
| Component | Weight | DD Estimate | Weight | DD Estimate |
| Dependent Variable | ln(Identity Theft) | ln(Identity Theft) | ln(Fraud) | ln(Fraud) |
|  |  |  |  |  |
| Early T vs Late C | 0.171 | -0.09 | 0.171 | 0.04 |
| Later T v Early C | 0.049 | -0.122 | 0.049 | 0.027 |
| Treated v Never | 0.78 | -0.026 | 0.78 | -0.106 |

**S6 Exhibit A5:** Sample Eliminating non-2014 Expansion

|  | (3) | (4) |
| --- | --- | --- |
| Estimator | PPML | PPML |
| Dependent Variable | Identity Theft | Fraud |
|  |  |  |
| Treatment | -0.0539 | -0.263*** |
|  | (0.0473) | (0.0564) |
|  |  |  |
| State FE | Yes | Yes |
| Year FE | Yes | Yes |
| Observations | 570 | 570 |
| Number of Groups | 38 | 38 |
| Robust standard errors in parentheses (clustered on state)  *** p<0.01, ** p<0.05, * p<0.1 | | |

**S7 Exhibit A6:** Average effect of ACA expansion on logged count of identity theft by year relative to expansion.


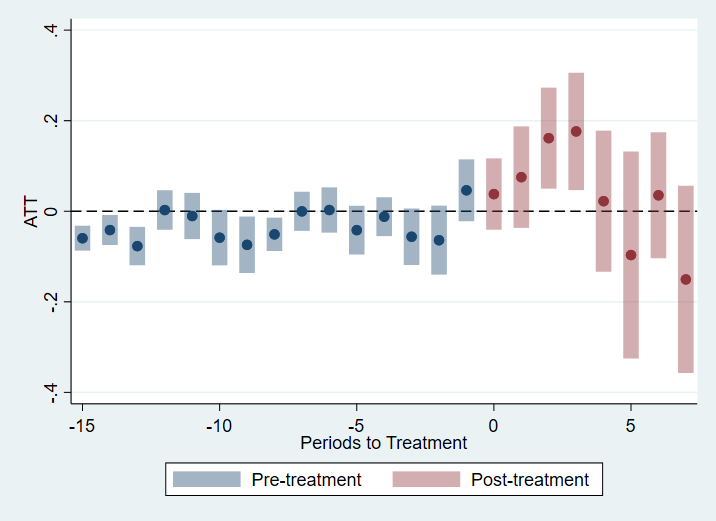


**S8 Exhibit A7:** Average effect of ACA expansion on logged count of fraud by year relative to expansion.


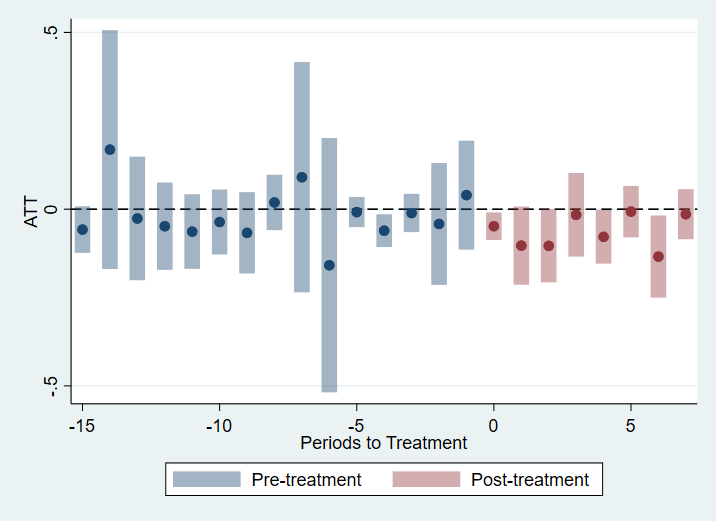

Supplement: S1 File — (DOCX) [file pone.0307015.s001.docx]
